# Elastofibroma dorsi: case report and review of the literature

**DOI:** 10.11604/pamj.2017.28.34.13675

**Published:** 2017-09-14

**Authors:** Basma Karrakchou, Youssef Yaikoubi, Mohamed Said Chairi, Abdelouahed Jalil

**Affiliations:** 1 Surgical Oncology Department 1, National Institute of Oncology, University Mohamed V Rabat, Morocco

**Keywords:** Elastofibroma dorsi, benign tumor, scapula, pathology, surgery

## Abstract

Elastofibroma dorsi (ED) is an uncommon benign soft tissue tumor with an uncertain pathogenesis. It mostly occurs in the infrascapular region of elderly people with a female predominance. Typically bilateral, ED can also be unilateral. While many patients remain asymptomatic, ED can be responsible of a periscapular arch source of ache. The diagnosis of ED is set on magnetic resonance imaging, and the pathological study ensures the diagnosis after surgical excision and establishes the differential diagnosis with malignant neoplasic process. The prognosis is excellent with extremely rare recurrence cases. Herein we report a case of a 54-years-old woman with a bilateral painful ED. The diagnosis was based on clinical and MRI findings that revealed bilateral tumors. Surgery was decided due to the symptomatic nature of the tumors. Pathological study confirmed the diagnosis. The post operative course was uncomplicated. We update through a review of the literature aspects of the diagnostic and therapeutic care of Elastofibroma dorsi.

## Introduction

Elastofibroma dorsi (ED) was first described in 1959 by Jarvi and Saxen and reported in 1961 in the 12^th^histopathology Scandinavian congress [1, 2]. ED is an infrequent soft benign tissue tumor with a slow growing, representing 1-2% of all primary tumors of the chest wall [3]. It's typically located in the posterior thoracic wall regarding the tip of the scapula, more precisely between the serratus anterior, and the latissimus dorsi muscle [4, 5]. ED is usually found in elderly women (above 50 years) and manual workers, as a unilateral mass of the back. Bilateral cases are although being more common than previously. We report a case of typically located bilateral elastofibroma dorsi in a 54-years-old woman in view of its rarity. From this observation we present the clinical, the imaging, and the pathological aspects of ED and we report our therapeutic strategy. We finally provide a review of the literature.

## Patient and observation

**Figure 1:**
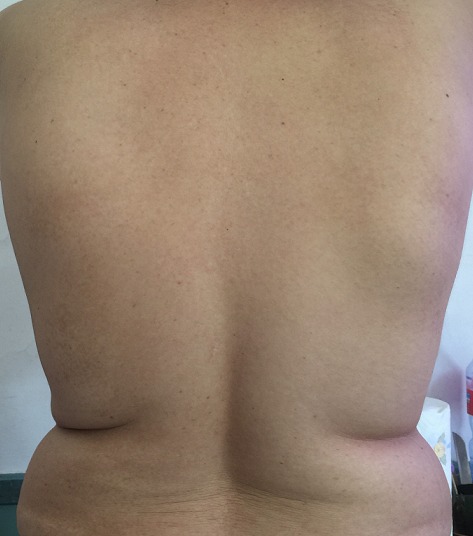
Front view showing the 2 scapular masses, more prominent in the right side and on abducting arms

**Figure 2:**
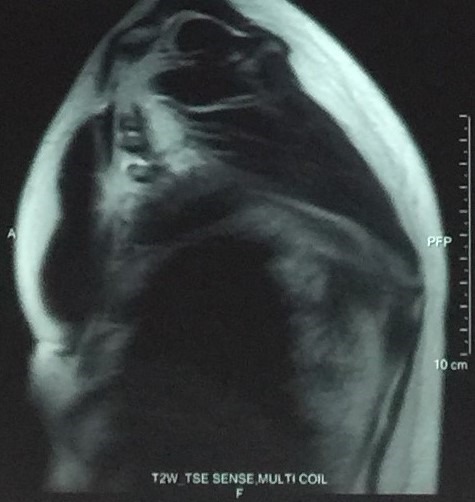
MRI image of elatofibroma dorsi (sagittal view)

**Figure 3:**
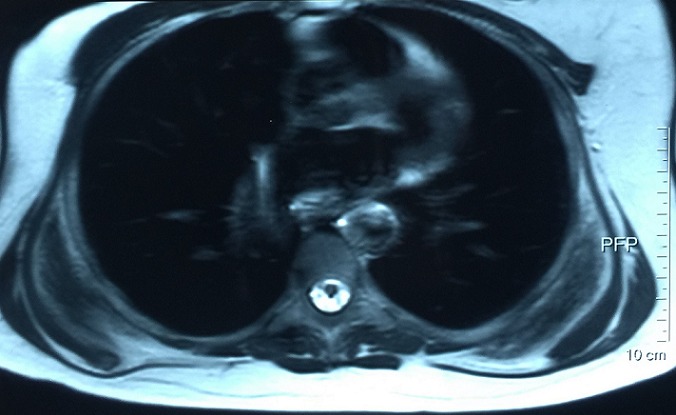
MRI image of bilateral elastofibroma dorsi (transversal view)

**Figure 4:**
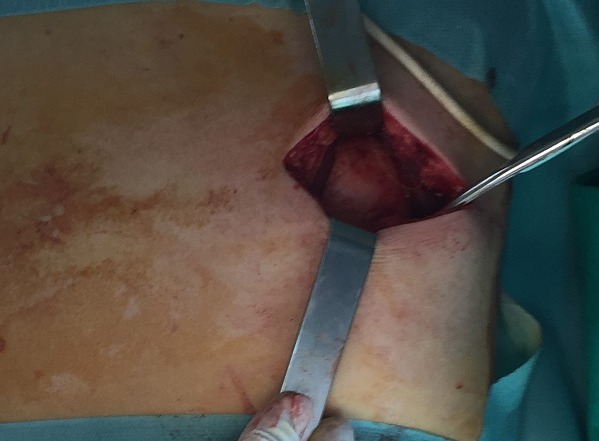
Intraoperative view of the subscapular mass

**Figure 5:**
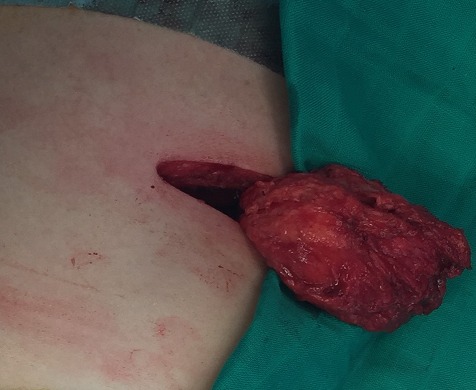
Surgical excision of the elastofibroma dorsi

**Figure 6:**
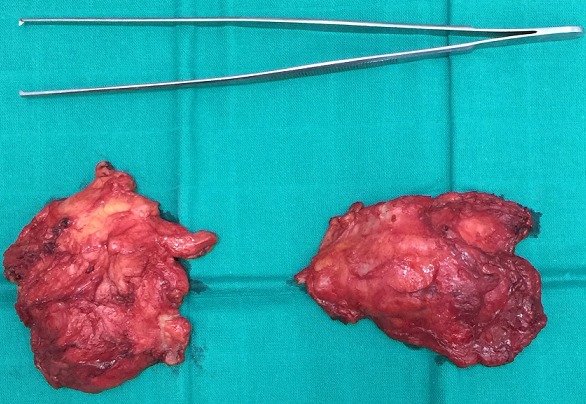
The two excised masses

A 54-years-old woman presented with a history of right mastectomy for a breast cancer 14 years previously, and radio-chemotherapy for squamous cells carcinoma of the cervix 12 years ago, and a cholecystectomy 5 years ago. She consulted for a winged right scapula since 1 year. In recent months the lesion had increased in size and caused discomfort and even pain in response to exercise. Physical examination revealed bilateral masses that became manifest on abducting and flexing arms (Figure 1). Both lesions were grossly rounded, of rubber-like consistency, not adhering to the skin but adhesive to deep structures, measuring about 9 cm in diameter, and slightly painful (the pain was reported mainly in the right side). Ultrasound showed a homogeneous well limited echogenic avascular structure measuring 67 x 30 mm. Magnetic resonance imaging set the diagnosis by revealing bilateral soft tissue tumors regarding the tip of the scapula, roughly symmetrical and poorly circumscribed with dimensions 77 x 21 mm in the right side and 83 x 21 mm in the left one. The masses consisted of elements with linear fibrillary signaling which intensity was equal to muscles in T1 and T2. Hypersignaling bands in T1 and T2 were also dispersed among them with intensity equal to fat. No signs of malignancy were observed, particularly absence of bone invasion. The MRI data were compatible with elastofibroma dorsi (Figure 2, Figure 3). Surgical excision was performed under general anesthesia on prone position and with abducted arms. An incision was made over the palpable masses and the ill-defined tumors were removed (Figure 4, Figure 5). Macroscopically, the lesions were non encapsulated, with whitish fibrous tracts alternating with yellowish tissue of fatty consistency (Figure 6). The histological study revealed a benign proliferation composed of collagen and elastic fibers, associated with some fibroblasts and fat cells. The elastic fibers were fragmented into globules or had a linear arrangement. The surgical excision was complete. The post operative course was without complications, and the patient was discharged 3 days after surgery.

## Discussion

Elastofibroma dorsi (ED) is an infrequent slow growing soft tissue benign tumor. It was defined in 2002 by the World Health Organization (WHO) of soft tissue tumors taxonomy as a benign fibroblastic/myofibroblastic tumor [5]. Nonetheless, most authors do not consider ED as a real tumor, but a hyperplasia of elastic tissue derived from fibroblasts due to chronic friction [6]. In reality, the pathogenesis of ED remains unsolved and is still open to controversy, but three etiological theories remain dominant. The first one suggests that chronic and repetitive mechanical stress leads to microtrauma, then to overproduction of elastic tissue from the stimulated fibroblasts. And the description of heavy manual worker men with repetitive labor was compatible with this theory [7, 8]. Thus, our clinical case seems to refute this thesis. Afterwards, ED tends to affect elderly women like our patient, which led to the second theory of reactive fibromatosis and secondary degeneration of elastic fibers due to vascular insufficiency [9]. At last, a third theory suggests a familial predisposition with an underlying enzymatic disorder or defect, reported by Fukuda and al [4, 7, 9, 10]. A double location of elastofibroma including the stomach and the scapula has also been described and is in favor of this last theory [11, 12].

Elastofibroma dorsi affects primarily the elderly over 55 years of age, with a mean age of 60 years [13]. Nevertheless, some cases of ED have been described also in children [14]. ED is found more frequently in women rather than men with a sex ratio Female/Man going from 5/4 to 13/1 [15]. Although ED is infrequent and with a slow growing, its diagnosis should be mentioned whenever a patient presents a discomfort in response to scapula movement. Indeed, the conception that ED is a very rare lesion seems to be unjustified probably because of the small size, the asymptomatic, and the benign nature of the lesion. As consequence, ED is usually accidentally found in CT/MRI imaging or when surgery is performed for other reasons [16]. Regarding the localization of elastofibroma, the vast majority of them are located in the subscapular and infrascapular region between the thoracic wall, serratus anterior, and latissimus dorsi muscles [1]. Unusual locations are also described, including mediastinum, stomach, peritoneum, ischial tuberosities, olecranon, deltoid muscle, intraspinal spaces, and foot [17, 18]. Bilateral involvement occurs only in 10% to 66% of cases, and is usually asynchronous [3, 5]. In our case, it was a typical dorsal elastofibroma in an elderly woman, but bilateral and asynchronous. Clinically, Elastofibroma dorsi is asymptomatic in 50% [4, 5, 7]. Symptoms depend on the site and the size of the lesion. When symptoms occur, they consist in discomfort or stiffness in shoulder abduction [19, 20]. A painful scapula is only observed in 10%. And a neurological involvement of the upper limb may be exceptionally observed, suggesting cervico-brachial neuralgia [21].

The physical examination reveals in typical ED a solid mass, of variable size (4-12 cm), adherent to the deep layers but non adherent to the skin, more prominent in abducting the arm, painless, usually in the right side[15] but may be present as well on the opposite shoulder, often smaller and silent [8, 10, 13, 22]. Radiological investigation is performed once the diagnosis is suspected. Chest X-ray shows unspecific images of elevation of the scapula and an enlargement of the scapulothoracic space. An interscapulothoracic opacity can be observed, but without bone lysis or associated calcification [19]. Ultrasound, CT and MRI imagings demonstrate the typical characteristics of ED which are collagen or elastic fibers in the fatty background [6]. Indeed, ultrasound examination shows an alternating of hypoechogenic and hyperechogenic striations similar to muscle and parallel to its major axis [23, 24]. CT scan shows a non homogeneous mal limited mass with density similar to muscles, including areas of lower density secondary to fat [6]. Finally, the MRI remains the gold standard examination for diagnosis. In fact, it shows heterogeneous well defined mass revealing two different T1 signals, one of an intermediate intensity equivalent to skeletal muscles signals, and the second of high intensity representing fat imprisoned within the mass. In T2, an increase in the signal intensity is observed. Injection of gadolinium doesn't enhance the signal [17, 25]. MRI imaging provides also specific features to establish differential diagnosis between sarcoma, liposarcoma, hemangioma, hematoma, lipoma and several other lesions. In our patient, the diagnosis was set based on clinical and MRI findings which were compatible with the literature's data.

Biopsy is performed only when diagnosis can't be set in front of untypical MRI findings, it provides then confirmation of ED [26]. Fine needle aspiration can also be used but is inadequate to get a representative tissue specimen. Macroscopically, elastofibroma is a non-encapsulated mass, poorly defined, measuring 2 to 15 cm, with a rubber consistency, which the cut surface exhibits white fibrous tissue with interposing small areas of yellow fat [5]. Histological examination describes a collagenous tissue, mixed with eosinophilic elastic fibers fragmented into disks or globules, associated with mature fat cells, as described in our case. It's important to notice that there is no atypia or mitotic activity, which distinguishes ED from other pseudotumors and neoplasms [4, 5]. Treatment of elastofibroma dorsi is provided only in symptomatic painful forms, or when the diagnosis is doubtful. It consists in complete surgical excision of the mass with curative marginal resection [13]. In asymptomatic lesions, clinical follow up proves to be sufficient. For some authors however, surgery is needed whenever the greater diameter is over 5 cm despite of the absence of symptoms [19, 20]. In our patient, we performed surgery due to the symptomatic aspect of the lesion, and the excision was complete. The postoperative course is usually simple, the most frequent complication remains hematoma due to the hypervascularisation of the subscapular region [4]. Thus, our patient didn't have any complication. The prognosis is excellent, extremely low recurrence cases have been described due to incomplete excision. But no malignant transformation has been reported [10].

## Conclusion

Elastofibroma dorsi is an infrequent benign soft tissue tumor which frequently occurs in the subscapular region of elderly women. Although it is frequently asymptomatic, it can be responsible of discomfort and pain in arm mobilization. MRI is the gold standard examination for diagnosis. Biopsy is performed only in untypical ED to rule out a malignant tumor diagnosis. Surgical excision is the therapeutic option for symptomatic patients only. Pathological study confirms the diagnosis after surgery. Finally, our experience with this case report is in accordance with the literature.

## Competing interests

The authors declare no competing interests.
